# Differences and Similarities in Motives to Decrease Drinking, and to Drink in General Between Former and Current Heavy Drinkers—Implications for Changing Own Drinking Behaviour

**DOI:** 10.3389/fpsyg.2021.734350

**Published:** 2022-01-12

**Authors:** Magdalena Rowicka

**Affiliations:** Department of Social Sciences, Institute of Psychology, The Maria Grzegorzewska University, Warsaw, Poland

**Keywords:** drinking motives, alcohol dependence, heavy drinking, low risk drinking, motives to decrease drinking

## Abstract

The evidence on why people initiate or cease drinking is vast; however, little is known regarding why people change their frequency and amount of drinking from intense (heavy or dependent drinking) to recreational (with little risk). Therefore, the purpose of this study was to investigate how drinking motives and motives to decrease drinking differ between former heavy drinkers (problematic and dependent), current dependent, and current recreational drinkers. Data were obtained from four groups of individuals (*n* = 263) using alcohol with different severity. The participants were Polish young adults aged between 18 and 35 years. About 53% of the sample were women. The Alcohol Use Disorder Identification Test (AUDIT) was used to assess the level of drinking; the Drinking Motive Questionnaire-Revised Short Form (DMQ-R SF) was used to assess drinking motives (social, coping, enhancement, and conformity). The reasons for abstaining and limiting drinking (RALD) instrument was used to assess the RALD. Additionally, a set of questions regarding motives to decrease drinking were analysed. The results show that differences were observed between the investigated groups: the current dependent group scored significantly higher on all the dimensions of drinking motives than the current low-risk group and significantly higher on coping, social, and enhancement motives than former heavy drinkers (both groups). The two groups of former heavy drinkers did not differ from each other on drinking motives. The investigated groups differed on the motives to reduce drinking—low-risk users scored the lowest on all the motives, whereas current dependent—the highest. The differences in motives to decrease drinking between current-depended and former heavy drinkers indicate which motives can be associated with the prevention strategies, programmes, and therapeutic approaches.

## Introduction

Alcohol use initiation usually takes place in adolescence (Johnston et al., 2003) and is followed by excessive drinking episodes more often in late adolescence and early adulthood (at ages 15–24 years) than at any other developmental stage (Gmel et al., 2003; RARHA, 2016). An early initiation can increase the risks for alcohol problems later in life (McCambridge et al., 2011; Kuntsche et al., 2017). The evidence on why people initiate alcohol consumption and engage in drinking, in general, is vast and mainly related to drinking motives. Research demonstrates that drinking motives predict alcohol use better than alcohol expectancies (Cronin, 1997) and contribute significantly in explaining the alcohol use variance together with situational factors or local norms (Kairouz et al., 2002). It is, therefore, crucial to understand what motivates people to drink and how those motivations differ between recreational and heavy alcohol users. Such knowledge could be further applied in various prevention and harm reduction interventions (Labrie et al., 2011).

Drinking motives can be characterised as psychological reasons for drinking and are based on the assumption that drinking satisfies different needs and serves different functions (Cox and Klinger, 1988; Cooper, 1994). The most common and influential model (Cooper, 1994) proposes that drinking motives can be described on two dimensions: the former one is related to reinforcement sought from alcohol use (positive or negative), and the latter one is related to the expected consequences of alcohol consumption (internal or external). The model proposes four drinking motives: coping with negative affect (negative and internal, e.g., “to forget your worries”), conformity with others (negative, external, e.g., “not to be left out”), enhancement of positive affect (positive and internal, e.g., “because it’s fun”), and social experience (positive and external, e.g., “to celebrate a special occasion with a friend”).

Studies show that different intensity of drinking is related to different motives; for example, heavy drinking is related to stress and need for coping (coping motive), as well as socialising in case of heavy peer drinking (social or conformity motive) (Abbey et al., 1993; Carpenter and Hasin, 1998). The coping motive has been repeatedly associated with negative consequences and heavy drinking (Kuntsche et al., 2005; Merrill et al., 2014). Therefore, it is expected that heavy alcohol users, such as dependent users, will score significantly higher on coping motives. Less is known regarding social and enhancement motives. A prior research was inconclusive—on the one hand, positive reinforcement motives (enhancement and social) are related to patterns of alcohol use but might be unrelated to drinking problems; on the other, they are related to both (predicting problems related to drinking) (Cooper, 1994; Cooper et al., 1995; Kuntsche et al., 2005; Littlefield et al., 2010; Hauck-Filho et al., 2012). It is important to note that the two motives—coping and enhancement—are related to affect (the former one—negative, and the latter—positive) and regulation *via* drinking, especially among young adults (Read et al., 2003). Some studies showed that enhancement motives are related to heavy drinking, whereas coping motives were related to adverse consequences (both related and unrelated to heavy drinking) (Kuntsche et al., 2005, 2014; Kuntsche and Kuntsche, 2009; Merrill and Read, 2010). Finally, conformity motives were associated with lower and not higher alcohol use (Kuntsche et al., 2014).

The evidence on why people initiate alcohol consumption or drink in general is vast; however, little is known why people change their frequency and amount of drinking from intense (heavy or dependent drinking) to recreational (with little risk). Therefore, it is essential to understand why people reduce their drinking—is it due to negative outcome expectancies (beliefs about the negative consequences of drinking) or due to abstention or drinking-reduction motives. Empirical evidence on the relationship between negative alcohol expectancies and consumption is mixed (Adams and McNeil, 1991; Jones et al., 2001), and little is known about the abstention motives and motives to decrease or limit drinking. The concept of motives or reasons for abstaining and limiting drinking (RALD) is under-research; however, numerous instruments measure RALD dating back to the 1970s (Epler et al., 2009). As a result, no standard RALD measure or instrument is accepted in the field, and researchers employ different approaches. Factorial structure of RALD is not a standard, as well—researchers conducted studies using one or two RALD factors (Nagoshi et al., 1994) and three and more (Collins et al., 2000; Epler et al., 2009). The full factorial structure of RALD comprises religious/moral considerations, a desire to maintain personal control, upbringing, concerns regarding expense, and desire to avoid adverse consequences (Epler et al., 2009). The research shows that the first three factors are negatively associated with drinking and the latter—positively (Collins et al., 2000). On the other hand, it was shown that health-related RALD (such as, health concerns) are associated with limiting own drinking (Wisk et al., 2020).

Though scarce, evidence showing the relationship between drinking motives, drinking severity, RALD and drinking behaviour is available. Similarly, the empirical evidence suggesting how drinking motives differ between dependent and low-risk alcohol users are rare. There is very little known about the difference between various alcohol users and their RALD.

Therefore, the purpose of this study was to investigate how drinking motives and motives to decrease drinking differ between former heavy drinkers (problematic and dependent), current dependent, and current low-risk drinkers.

Based on the literature, it is hypothesised that (1) current dependent users will score significantly higher on coping and enhancement motives than current low-risk users; (2) there will be no or slight difference between current dependent and current low-risk users with regard to social motives.

Furthermore, it is hypothesised that (3) the three heavy user groups will score higher on coping motives than current low-risk users. Further, it is hypothesised that (4) there will be differences in conformity and enhancement motives between the investigated groups, but the direction is undetermined. Since enhancement motivation is related to hedonistic motives and the introduction of positive mood states, it could be higher among low-risk users; on the other hand, when viewed as a compensation mechanism, it could be coupled with coping mechanism acting as positive expectancies and therefore, scoring higher among heavy alcohol users. Additionally, it is hypothesised that (5) coping, conformity, and enhancement motive will predict problem related to drinking [in terms of the Alcohol Use Disorder Identification Test (AUDIT) score], and social motives will either predict problem of drinking slightly or insignificantly. Finally, it is hypothesised that (6) motives related to religious/moral considerations, maintaining personal control, and upbringing will be associated negatively with drinking, whereas motives related to avoiding adverse consequences—positively. It is expected that (7) current dependent users will score higher on all motives than current low-risk users. There is insufficient evidence to expect whether former heavy users will score similarly to the current dependent users or current low-risk users. On the one hand, the former heavy user groups shared heavy drinking experiences with current dependent users; on the other hand, the two former heavy users decreased their drinking (some due to therapy) and currently keep on drinking but with low risk.

## Materials and Methods

### Participants

The participants were Polish young adults aged 18 and 35 years (*M* = 29.31; SD = 4.28) consuming alcohol with various intensities and severity. There were three inclusion criteria: (1) being young adult (between 18 and 35 years old); (2) drinking alcohol within the last 12 months prior to the study; and (3) being classified into one of the four groups: (1) current low risk: drinking alcohol currently but with low risk [as indicated in the AUDIT (Saunders et al., 1993; Babor et al., 2001)] and with no history of alcohol abuse (*n*1 = 78); (2) current dependent: being alcohol dependent—drinking alcohol in the last 12 months, receiving the diagnosis of alcohol dependence, and not being in treatment for a period longer than 3 months prior to the participation in the study; additionally, individuals who were classified into this group scored above 20 points in the AUDIT (Saunders et al., 1993; Babor et al., 2001) (*n*2 = 82); (3) former heavy users current low risk: drinking currently (within the last 12 month) with low risk [up to 8 points in the AUDIT (Saunders et al., 1993; Babor et al., 2001)] but with a history of alcohol abuse in the past (*n*3 = 46) (but no diagnosis); and (4) former dependent current low risk: drinking currently (within the last 12 month) with low risk [up to 8 points in the AUDIT (Saunders et al., 1993; Babor et al., 2001)] but being diagnosed with dependence in the past (*n*4 = 56).

The exclusion criteria included dependence treatment (different than alcohol), abuse of other substances, as well as and prescribed opioid use more than 10 times in a lifetime (for more details, see Rosenkranz et al., 2019).

Within 262 participants, 49% were male, and 51% were female. Sex distribution between the four investigated groups was equal [chi^2^(3) = 4.61; *p* = 0.20]. The majority of the participants had at least high school education (90.5%), only 2% had primary education, 8% had vocational education, 38% had high school education, and 53% had higher education. There were minor differences in education structure between the four investigated groups [chi^2^(9) = 19.68; *p* = 0.020] related to a slightly lower level of education in the two dependent groups (currently and in the past). The places of residence of participants were varied, but their distribution did not differ between the four investigated groups [chi^2^(21) = 24.77; *p* = 0.257]. About 37% of the participants were single, and a further 40% were married. Approximately 19% of the participants were in informal relationships, and 4% were disordered. The four investigated groups differed significantly with respect to marital status [chi^2^(9) = 40.34; *p* < 0.001]—in Group 1 (currently low-risk drinking with no history of alcohol abuse), there was significantly more participants in informal relationships than in any other group and significantly fewer married participants than in Group 2 (currently dependent) and Group 3 (problematic in the past). The descriptive statistics are presented in Table 1.

**TABLE 1:** Sociodemographic variables in the total sample and the four investigated groups.

Sociodemographic variables	Current low risk (Group 1)		Current dependent (Group 2)		Former heavy user current low risk (Group 3)		Former dependent current low risk (Group 4)		Total group	
					
	N	%	n	%	n	%	n	%	n	%
**Sex**										
Males	34	43.6	48	58.5	20	43.5	26	46.4	128	48.9
Females	44	56.4	34	41.5	26	56.5	30	53.6	134	51.1
**Education**										
Primary	1	1.3	2	2.4	0	0.0	2	3.6	5	1.9
Vocational	3	3.8	7	8.5	4	8.7	6	10.7	20	7.6
High school	20	25.6	39	47.6	14	30.4	26	46.4	99	37.8
Higher	54	69.2	34	41.5	28	60.9	22	39.3	138	52.7
**Place of residence**										
Village	10	12.8	15	18.3	6	13.0	6	10.7	37	14.1
City up to 19,000 residents	7	9.0	7	8.5	6	13.0	8	14.3	28	10.7
City 20,000–49,999 residents	7	9.0	12	14.6	2	4.3	4	7.1	25	9.5
City 50,000–99,999 residents	7	9.0	12	14.6	8	17.4	6	10.7	33	12.6
City 100,000–199,999 residents	9	11.5	9	11.0	4	8.7	10	17.9	32	12.2
City 200,000–499,999 residents	9	11.5	11	13.4	8	17.4	14	25.0	42	16.0
City above 500,000 residents	23	29.5	11	13.4	8	17.4	6	10.7	48	18.3
Warsaw	6	7.7	5	6.1	4	8.7	2	3.6	17	6.5
**Marital status**										
Single	26	33.3	28	34.1	16	34.8	26	46.4	96	36.6
Married	22	28.2	41	50.0	26	56.5	16	28.6	105	40.1
Divorced	1	1.3	4	4.9	0	0.0	6	10.7	11	4.2
Non-formal relationship	29	37.2	9	11.0	4	8.7	8	14.3	50	19.1
**Employment status**										
Employed	63	81	70	85	38	83	44	79	215	82
Retired	0	0	1	1	0	0	2	4	3	1
Unemployed	2	3	8	10	2	4	6	11	18	7
Student	10	13	0	0	2	4	4	7	16	6
Not working (other reason than unemployed)	3	4	3	4	4	9	0	0	10	4

### Materials

Participants were classified into one of the investigated groups based on a set of questions regarding their alcohol dependence (in the last 12 months and lifetime), participation in treatment (in the last 12 months and lifetime), and current drinking (in the last 12 months). To confirm current dependence and current low-risk drinking, the AUDIT (Saunders et al., 1993; Babor et al., 2001) was administered. To classify the participants into Group 3 (former heavy users and current low risk), a set of questions derived from the AUDIT was used but regarding a year in the past (not last 12 months) with the most intensive drinking pattern. The participants classified into Group 3 scored above 16 points (mainly due to questions 1–4 and 7–8, drank above 40/60 ml of alcohol per day, and have never been diagnosed with alcohol dependence).

Additionally, the demographic questions regarding age and sex were asked, as well as regarding receiving a diagnosis recently or in the past, and any alcohol treatment experience.

The Drinking Motive Questionnaire-Revised Short Form (DMQ-R SF) (Cooper, 1994) was used to assess drinking motives (social, coping, enhancement, and conformity).

#### Drinking Motive Questionnaire

The factorial structure of the DMQ-R SF was investigated. The assumed four-factor structure yielded satisfactory results based on the criteria for global fit indexes (Browne and Cudeck, 1993; Jöreskog and Sörbom, 1993; Hu and Bentler, 1999; Kline, 2005). Further, internal consistency reliabilities (Cronbach alpha) were calculated for each of the factors. The internal consistency reliabilities of three factors were satisfactory (Cronbach’s alpha: 0.77 for coping, 0.814 for conformity, and 0.758 for social) and less satisfactory for one factor (Cronbach’s alpha 0.56 for enhancement). To investigate the low alpha in one of the original factors of the DMQ-R SF, an exploratory factor analysis (EFA) was conducted. The EFA resulted in a three-dimensional structure (explaining 62% of variance). The three-factor structure yield satisfactory results based on the criteria for global fit indexes (Browne and Cudeck, 1993; Jöreskog and Sörbom, 1993; Hu and Bentler, 1999; Kline, 2005), as well as convergent and discriminant validity indicators (Hu and Bentler, 1999; Supplementary Tables 1, 2). However, for clarity purposes, only the original four-factor model was presented in the article.

#### Motives to Decrease Drinking

Motives to decrease drinking derived from the qualitative arm of the study. An exploratory and confirmatory analysis structured them into: negative consequences (e.g., to avoid adverse psychological consequences because of regret of having done something under the influence of alcohol), social (e.g., familial disapproval), neglect of other duties (e.g., school and work), and loss of control (Supplementary Table 3).

#### Reasons for Abstaining and Limiting Drinking

The RALD were employed (Epler et al., 2009) and were tested with EFA to establish their factorial structure. The EFA showed that three factors explain 78.5% of the variance. The factors were named: (1) loss of control and negative consequences, (2) outgrowing, and (3) avoidance of negative physical and psychological consequences. The exact working and factorial loadings of the statement are presented in Supplementary Table 4.

### Procedure

The reported results derive from a mixed-method study conducted in the years 2019 and 2020. First, qualitative semi-structured interviews were conducted to identify motivational and individual aspects that might change the drinking trajectory. The results informed the quantitative arm of the study that the substantial focus was on investigating the motives to drink and reduce drinking. The quantitative part was conducted partially in person and partially *via* phone/skype and Internet platform [due to coronavirus disease 2019 (COVID-19) restrictions]. Participants in the two arms of the study were not redundant. Participants were awarded a voucher for an approximate value of 6 euros.

## Results

Before the hypotheses were tested, the descriptive statistics were analysed (Tables 2, 3). Analysis of Skewness and Kurtosis for the total sample (Table 2) and subsamples (Table 3) showed that normal distribution could be assumed for three motives (with an exception for conformity) (Ghasemi and Zahediasl, 2012). To deal with deviation from the normal distribution, bootstrapping was employed, and multivariate ANOVA (MANOVA) and Pearson’s correlations were conducted. Finally, the regression analysis was conducted with the prior investigation of assumptions. The distribution of residuals in the regression analysis was not deviating from normal, and additionally, Mahalanobis distance analysis did not reveal any outliers (Tabachnick and Fidell, 2007).

**TABLE 2:** Descriptive statistics for motives, Alcohol Use Disorder Identification Test (AUDIT), and age in the total sample.

	*N*	Min	Max	Mean	SD	Skewness	Skewness’ SE	Kurtosis	Kurtosis’ SE
Coping	263	1.00	3.00	2.00	0.54	0.019	0.150	−0.471	0.299
Conformity	263	1.00	3.00	1.64	0.60	0.751	0.150	−0.299	0.299
Social	263	1.00	3.00	2.23	0.49	−0.228	0.150	−0.095	0.299
Enhancement	263	1.00	3.00	1.95	0.48	0.178	0.150	−0.339	0.299
AUDIT	263	0	40	12.93	9.47	0.814	0.150	−0.179	0.299
Age	263	18	35	29.29	4.29	−0.531	0.150	−0.664	0.299

**TABLE 3:** Descriptive statistics for motives, AUDIT, and age across groups.

		*N*	Minimum	Maksimum	Średnia	Odchylenie standardowe	Skewness	SE Skewness	Kurt	SE Kur
Current low risk (Group 1)	Coping	79	1.00	3.00	1.8523	0.46455	0.229	0.271	−0.002	0.535
	Conformity	79	1.00	3.00	1.4473	0.49753	1.122	0.271	0.955	0.535
	Social	79	1.00	3.00	2.0506	0.51499	−0.239	0.271	−0.263	0.535
	Enhancement	79	1.00	3.00	1.6793	0.46053	0.679	0.271	0.510	0.535
	AUDIT	79	0	7	4.08	1.591	−0.323	0.271	−0.155	0.535
	Age	79	18	35	28.78	4.590	−0.362	0.271	−0.979	0.535
Current dependent (Group 2)	Coping	82	1.00	3.00	2.2520	0.51203	−0.122	0.266	−0.312	0.526
	Conformity	82	1.00	3.00	2.0772	0.59097	0.129	0.266	−0.816	0.526
	Social	82	1.33	3.00	2.3577	0.45294	0.016	0.266	−0.914	0.526
	Enhancement	82	1.00	3.00	2.2480	0.46793	−0.050	0.266	−0.545	0.526
	AUDIT	82	20	40	25.21	5.452	1.327	0.266	1.006	0.526
	Age	82	21	35	30.29	3.851	−0.674	0.266	−0.481	0.526
Former heavy user current low risk (Group 3)	Coping	46	1.00	2.67	1.8406	0.45400	−0.158	0.350	−0.435	0.688
	Conformity	46	1.00	2.00	1.2899	0.37587	1.045	0.350	−0.355	0.688
	Social	46	1.67	3.00	2.2754	0.38041	0.732	0.350	−0.548	0.688
	Enhancement	46	1.33	2.67	1.9130	0.34737	0.063	0.350	−0.390	0.688
	AUDIT	46	3	14	9.04	3.204	−0.185	0.350	−0.921	0.688
	Age	46	19	35	29.78	4.033	−1.292	0.350	1.588	0.688
Former dependent current low risk (Group 4)	Coping	56	1.00	3.00	1.9881	0.62592	−0.186	0.319	−0.954	0.628
	Conformity	56	1.00	3.00	1.5357	0.53439	0.897	0.319	0.187	0.628
	Social	56	1.00	3.00	2.2500	0.50553	−0.466	0.319	0.126	0.628
	Enhancement	56	1.00	2.67	1.9286	0.39550	−0.380	0.319	−0.411	0.628
	AUDIT	56	2	15	10.64	4.002	−0.681	0.319	−0.744	0.628
	Age	56	20	35	28.11	4.393	−0.016	0.319	−1.100	0.628

### Drinking Motives

To test hypotheses 1–4, two sets of multivariate analyses of variance with bootstrapping were conducted. Hypothesis 1 assumed that current dependent users will score significantly higher on coping and enhancement motives than current low-risk users; hypothesis assumed no differences between the current dependent and current low-risk users with regard to social motives; hypothesis 3 assumed that the three heavy user groups will score higher on coping motives than current low-risk users; and hypothesis 4 assumed there will be differences in conformity and enhancement motives between the investigated groups.

The analysis conducted for the drinking motives (four-factor model) showed differences in all four motives, Wilk’s Lambda = 0.63; *F*(4,12) = 674.96; *p* < 0.001; and partial eta-squared = 0.143. Current dependent users (Group 2) scored significantly higher on coping and conformity motives compared with all other groups (current low risk and both former heavy/current low risk); the effect size was stronger for conformity (partial eta-squared = 0.262) than for coping (partial eta-squared = 0.109). This supports hypothesis 1 and goes beyond since only differences between current dependent users (Group 2) and current low-risk users (Group 1) were assumed, together with only two motives). Current dependent users (Group 2) scored higher on enhancement motive than formers users (Groups 3 and 4) (no distinction was found between the two former groups) and current low-risk users (Group 1), as hypothesized (H1, H3 and H4). Additionally, both the former user groups (Groups 3 and 4) scored higher than the current low-risk group (Group 1). Finally, Current dependent users (Group 2) scored significantly higher on social motive than current low-risk users (Group 1) (contrary to what was hypothesized – lack of differences between these two groups, H2) (Table 4).

**TABLE 4:** Drinking motives (four-factorial)—group comparison.

Motives	Group	Mean	SE	95% LLCI	95% ULCI	*F* (3,261)	*p*-Value	Partial eta-squared
Coping	Current low risk (Group 1)	1.84*^b^*	0.058	1.73	1.96	10.51	<0.001	0.109
	Current dependent (Group 2)	2.25*^a^*	0.057	2.14	2.36			
	Former heavy user current low risk (Group 3)	1.84*^b^*	0.076	1.69	1.99			
	Former dependent current low risk (Group 4)	1.99*^b^*	0.069	1.85	2.12			
Conformity	Current low risk (Group 1)	1.45*^b^*	0.059	1.34	1.57	30.52	0.001	0.262
	Current dependent (Group 2)	2.08*^a^*	0.057	1.96	2.19			
	Former heavy user current low risk (Group 3)	1.29*^b^*	0.077	1.14	1.44			
	Former dependent current low risk (Group 4)	1.54*^b^*	0.069	1.40	1.67			
Social	Current low risk (Group 1)	2.06*^b^*	0.053	1.96	2.17	5.45	<0.001	0.060
	Current dependent (Group 2)	2.36*^a^*	0.052	2.26	2.46			
	Former heavy user current low risk (Group 3)	2.28	0.069	2.14	2.41			
	Former dependent current low risk (Group 4)	2.25	0.063	2.13	2.37			
Enhancement	Current low risk (Group 1)	1.69*^b^*	0.049	1.59	1.78	22.86	<0.001	0.210
	Current dependent (Group 2)	2.25*^a^*	0.048	2.15	2.34			
	Former heavy user current low risk (Group 3)	1.91*^c^*	0.063	1.79	2.04			
	Former dependent current low risk (Group 4)	1.93*^c^*	0.058	1.81	2.04			

*SE, standard error; LLCI, lower lever confidence interval; ULCI, upper-level confidence interval. For between-group comparison, Bonferroni post hoc was chosen due to no differences in invariances. Different indices (a–c) indicate differences in means between the groups.*

To test hypothesis 5 (assuming that only coping, conformity, and enhancement motives will not predict the score in AUDIT), a regression model was used. In the model, the score in AUDIT was the explained variable, and the four motives were predictors (Table 5). A correlation analysis showed that all the motives are associated with drinking severity (Table 6).

**TABLE 5:** A regression model with an AUDIT score as an explained variable and motives to drink as predictors (four-factor model).

	*B*	SE	Beta	*t*	*p*	*F* (4,261)	*p*	Adj *R*^2^
Coping	2.469	1.011	0.141	2.443	0.015	43.95	<0.001	0.397
Conformity	4.994	0.859	0.317	5.815	0.000			
Social	−0.409	1.176	−0.021	−0.348	0.728			
Enhancement	6.810	1.291	0.347	5.276	0.000			

**TABLE 6:** Pearson’s correlation with bootstrapping of AUDIT and motives (*N* = 262).

		Coping	Conformity	Social	Enhancement
AUDIT	Pearson’s r	0.433***	0.515***	0.331***	0.544***
	95% LLCI	0.326	0.400	0.209	0.433
	95% ULCI	0.538	0.618	0.434	0.640
Coping	Pearson’s r		0.385***	0.404***	0.513***
	95% LLCI		0.258	0.292	0.400
	95% ULCI		0.498	0.516	0.615
Conformity	Pearson’s r			0.295***	0.431***
	95% LLCI			0.162	0.316
	95% ULCI			0.422	0.542
Social	Pearson’s r				0.581***
	95% LLCI				0.494
	95% ULCI				0.661

***p < 0.001.

A regression analysis showed that three out of four motives predict severity of drinking significantly—coping, conformity, and enhancement (as predicted in the hypothesis 5). Social motive did not predict the severity of drinking. Moreover, all three motives positively predict the severity of drinking—the higher the motive, the higher the serenity of drinking. Additionally, conformity and enhancement predicted the severity of drinking moderately (beta: 0.317 and 0.347), whereas coping—in a relatively small manner (beta = 0.107) (Table 5).

### Motives to Decrease Drinking

To test hypotheses 6 and 7, a set of multivariate analyses of variance was conducted. Hypothesis 6 assumed that motives related to religious/moral considerations, maintaining personal control, and upbringing will be associated negatively with drinking, whereas motives related to avoiding adverse consequences—positively. Hypothesis 7 assumed that current dependent users will score higher on all motives than current low-risk users.

The MANOVA showed the differences in all three motives to decrease drinking, Wilk’s Lambda = 0.623; *F*(9,623) = 14.86; *p* < 0.001; and partial eta-squared = 0.146. Current low-risk users (Group 1) scored significantly lower on all three motives than other groups (current dependent and former heavy users). Additionally, differences were observed in the “loss and negative consequences” motive—the two former heavy user groups (Groups 3 and 4) scored significantly higher than the current low-risk group (Group 1) but significantly lower than the current dependent group (Group 2). The current low-risk group (Group 1) scored significantly lower than others in the two remaining motives (Table 7).

**TABLE 7:** Multivariate ANOVA (MANOVA) for motives to decrease drinking across groups.

Motives	Groups	Mean	SE	95% LLCI	95% ULCI	*F*(3,261)	*p*-Value	Partial eta-squared
M2_loss	Current low risk (Group 1)	1.67*^a^*	0.103	1.47	1.88	42.366	<0.001	0.330
	Current dependent (Group 2)	3.23*^b^*	0.100	3.03	3.42			
	Former heavy user current low risk (Group 3)	2.00*^c^*	0.134	1.74	2.26			
	Former dependent current low risk (Group 4)	2.33*^c^*	0.121	2.09	2.57			
M2_outgrowing	Current low risk (Group 1)	2.31*^a^*	0.132	2.06	2.57	6.540	<0.001	0.071
	Current dependent (Group 2)	3.07*^b^*	0.129	2.81	3.32			
	Former heavy user current low risk (Group 3)	2.96*^b^*	0.172	2.62	3.30			
	Former dependent current low risk (Group 4)	2.95*^b^*	0.156	2.64	3.25			
M2_avoidance	Current low risk (Group 1)	2.22*^a^*	0.115	1.99	2.45	42.366	<0.001	0.122
	Current dependent (Group 2)	3.17*^b^*	0.113	2.95	3.39			
	Former heavy user current low risk (Group 3)	2.80*^b^*	0.150	2.51	3.10			
	Former dependent current low risk (Group 4)	2.88*^b^*	0.136	2.61	3.14			

*Different indices (a–c) indicate differences in means between the groups.*

### Reasons for Abstaining and Limiting Drinking

The MANOVA showed the differences in two out of three motives to decrease drinking (RALD): control [*F*(1,101) = 4.08; *p* = 0.046; and partial eta-squared = 0.039] and convictions [*F*(1,101) = 4.05; *p* = 0.047; and partial eta-squared = 0.039]. In both cases, formerly dependent users (Group 4) scored higher than formerly heavy users (Group 3).

Finally, the regression analysis was conducted to test hypothesis 7. The results showed that two out of three motives/reasons to limit drinking predicted severity of drinking: convictions (*B* = 2.260, *t* = −4.330, beta = 0.411; and *p* < 0.001) and adverse consequences (*B* = −1.37; *t* = −3.038; beta = −0.298; and *p* < 0.001). The model explained 18.4% of the variance of the severity of drinking.

## Discussion

The primary purpose of this study was to investigate the similarities and differences in drinking motives, as well as motives to decrease/reasons to limit drinking between four specific types of alcohol users—current low risk (with no prior history of alcohol abuse), current depended (in therapy not longer than for 3 months or not at all), formerly depended but currently, low risk, and formerly heavily drinking but currently drinking with low risk. Much attention was paid to differentiate between the current low-risk users with and without prior history of heavy alcohol use. In general, the results show that such a differentiation is important and, when lacking, may lead to unreliable results (lack of differences between different motives to drink between current low risk and current dependent users).

The main purpose of this study was not to adapt DMQ-R SF or RALD measure or to create a new one; however, to test the hypothesis, a set of exploratory and confirmatory analyses was conducted. As a result, two factorial structures of DMQ-R SF were tested, and a shorter one (with three factors) was proven to perform well or better (according to some parameters) on the investigated sample. Interestingly, two out of four original factors remained unchanged (coping and conformity), and the remaining two were shortened and merged into one (social enhancement). Not repeating the four-factor structure does not have to be treated as a mistake since some measurement approaches assume three factors (O’Hare, 1997, 2001; Labouvie and Bates, 2002). There are previous studies in which the four factorial structure was not confirmed (Martens et al., 2003; Mushquash et al., 2008). Kuntsche et al. (2005) analysed, available till the year 2005, instrument and theoretical conceptualisation in the field of drinking motives and concluded that the way motives are measured is high heterogeneous (from 10 to 40 items, from 2 up to 10 dimensions). It is, however, unclear whether the four factor DMQ-R SF model does not hold in this sample or does not hold in the sample of young adults (18–35 years) in general. The investigated sample in this study was specific and composed of four subsamples. Each of them is rather too small to perform a set of EFA and confirmatory factor analysis (CFA) on each of them separately. Additionally, the sample is not representative even though it was randomly chosen (from a panel). At this point, caution in interpretation is suggested and further investigation (e.g., in larger, though specific samples). For the purpose of hypothesis testing both, the original four factor model was tested, as well as the new, three factor model. The three factor model performed better in terms of psychometric properties and, hence, is valid.

This study provides an essential insight into the differences in motivational underpinnings of alcohol drinking and the limitation of drinking between various alcohol users. First, the investigated group comparison provided further empirical evidence to support the claim that there are differences between current low risk and current dependent users in their motives to drink. Heavy drinking (such as dependence) is related to coping and conformity (Cooper et al., 1995). Additionally, current dependent users scored higher on social and enhancement motives, which suggests that dependent alcohol users may drink on any occasion or that their drinking serves as compensation and, together with coping, they also have positive expectancies (therefore, social and enhancement motives). This aligns with the literature examining drinking motives among the dependent individuals (Kuntsche et al., 2007; Kuntsche and Kuendig, 2012; Lehavot et al., 2014; Simpson et al., 2014; Wicki et al., 2017).

Interestingly, the two former heavy user groups (Groups 3 and 4) did not differ from the current dependent group (Group 2) on three out of four motives: coping, conformity, and social, but differed on enhancement (in between current low risk and current dependent)—these results shed new light on how former heavy users are motivated to drink. The results showed that the main difference is related to enhancement motive—former heavy users drink currently with low risk, and hence their enhanced motive to drink is lower than in the case of dependent individuals, but all the other motives are at a similar level. Moreover, since other research showed that the enhancement and coping motives are associated with quantity and frequency of alcohol intake, the former heavy users are still at risk. Especially that coping motives are linked to the alcohol-related symptoms (Kuntsche et al., 2007; Kuntsche and Kuendig, 2012; Lehavot et al., 2014; Simpson et al., 2014; Wicki et al., 2017). The question remains whether heavy drinking individuals decreased their drinking due to decreased enhancement motive (e.g., drinking ceased being fun) or were exposed to any intervention that contributed to the decreasing positive expectancies related to drinking. This is particularly interesting since the two former heavy user groups were diagnosed with dependence and received treatment, whereas no one from the former heavy (not dependent) group received treatment. Either way, indicated prevention strategies and harm reduction interventions are promising in lowering the positive expectancies, supporting the development of various coping strategies, and correcting normative beliefs (Blevins and Stephens, 2016). It is essential to underline that the interventions which will not address (directly or indirectly) may fail.

Furthermore, the investigated motives were tested in the regression model. Contrary to some findings (Cooper et al., 1995), one avoidance motive (conformity) and one approach motive (enhancement) were moderately and positively associated with drinking severity. On the other hand, the social motive was not associated with drinking severity, and coping was associated positively but with a low magnitude. Researchers obtaining similar results suggest that the differences are due to cultural factors (Hauck-Filho et al., 2012), but they might also be related to age specificity (in this study, young adults were investigated) and group diversity (four groups). The sample was, however, too small to test the regression models separately for each group. It would also be troublesome due to the application of AUDIT to distinguish the groups (and AUDIT would serve as an explained variable). Such an undertaking would increase the homogeneity of the explained variable.

Furthermore, the lack of significance of social motive could be related to a poor fit of the four-factor DMQ-R SF model. If the effect is replicated, it may be explained by the cultural transition—alcohol use is not the only or the primary way to enhance social experiences. If so, these results would be supported by the work of Lee and Lewis—the researcher found that social motive is a strong predictor of alcohol abuse but in environments that support and encourage alcohol use (Lewis and Neighbors, 2006; Lee et al., 2007).

The investigated groups differed on the motives to reduce drinking—the main difference was between heavy users (both current and former) and current low-risk users. Heavy users scored significantly higher on all the three motives to reduce drinking, but the greatest difference was observed in the loss of control motive. Additionally, the loss of control differentiated current low-risk, current dependent, and former heavy users (with the last group scoring between the two current groups). This result is significant because it shows the proximity between current dependent and current low-risk users with heavy use in the past. It supports the results obtained in the field of drinking motives (Blevins and Stephens, 2016; Hammarberg et al., 2017). In addition, it is essential to notice that the motives to reduce drinking were measured in two ways—the first one was based on the results from the qualitative arm of this study and resulted in a proposed factorial structure; the second one was based on the RALD (Epler et al., 2009). This study did not confirm the original factorial structure, and a new one was employed to test the hypotheses.

Nevertheless, the new factors were similar to the original ones, with some items migrating between the factors. The results, however, showed that the two former heavy user groups differed on two out of three factors, namely, the control and the convictions. In both cases, former dependent users scored higher. Dependent individuals had higher convictions and loss of control motives to limit their drinking, and they were successful. They also had to limit their drinking significantly more than the other former group (from dependent to low-risk). This might implicate that in the case of successfully limiting own drinking, the stronger the motives to limit drinking are, the better the effect.

Furthermore, the regression analysis showed that convictions’ motives are positively and moderately associated with drinking severity, whereas adverse consequences motive—moderately but negatively. This result may seem to be reversed, but it is important to note that the regression analysis was run only on two former heavy—current low-risk users; hence the correlation between the motive and the alcohol use severity might be due to the group composition and homogeneity of alcohol severity results (AUDIT). Second, it may be that those who score a bit higher in the AUDIT belong to the former dependent group, and therefore a higher conviction played a protective role. If so, this result is congruent with the literature on the preventive aspects of religion (e.g., Lee et al., 2017). Furthermore, the negative association between the negative consequences and drinking severity is aligned with the literature—the more severe the negative consequences, the less severe the drinking is (Epler et al., 2009). However, whether the association is direct and causal or indirect remains unclear and should be treated as an indicator for alcohol-related problems (such as, unsuccessful attempts to limit drinking) (Epler et al., 2009).

The implications of investigating motives to decrease and reasons for liming drinking could be in the prevention domain. First, it was demonstrated that positive expectancies (e.g., in terms of enhancement motive) are challenged in the group of young adults (Epler et al., 2009). Some of the approaches were already implemented (Wood et al., 2007). As a result, apart from improving various skills (e.g., stress management), public health strategies could be focused on normative peer interventions to elevate the convictions (without prior experience of heavy drinking) (Lewis and Neighbors, 2006; Epler et al., 2009). Additionally, more insight into RALD may help assess the beliefs and even readiness to change stage, since strong religious and upbringing convictions are usually formed during childhood and could act as a protective factor, whereas experiencing negative consequences is related to an already existing problem.

Unequivocally, before further recommendations can be formed, further research investigating the relationship between the reasons and motives to limit/decrease drinking and drinking severity is needed.

## Limitations

The study is not free from limitations. It is important to note that primary purpose of the study was investigated in a cross-sectional design and not in a longitudinal one. The groups were carefully chosen, various inclusion and exclusion criteria were used, but the motives were investigated for the time being between the groups and not within the same group over time. Because of that, it cannot be stated with a higher level of certainty how the motives change together with the change of drinking patterns. Additionally, the group sizes were relatively small and, therefore, small effect sizes were possible for detection only for correlation and regression analysis but not for group comparison (in which case only medium to large effect sizes were possible for detection) (Malone et al., 2016).

Furthermore, the group participation was assessed based on the declarations from participants with no additional way to confirm the diagnosis. The only additional measure used was the AUDIT. Group 3 (former heavy user current low risk) was established with the most difficulty, and a modified AUDIT was used to enable it. It is important to note that the modification was not previously checked for its reliability and validity.

Finally, the RALD items yielded a different structure than the one obtained by Epler et al. (2009). Therefore, it is rather difficult to compare the results obtained in this study with the results obtained by other researchers. Other researchers, however, pointed out that the structure suggested by Epler et al. (2009) does not seem to be universal. Nevertheless, the stated hypothesis could not be verified due to the different factorial structure of the RALD.

## Data Availability Statement

The data analyzed in this study were obtained from PARPA, after the author designed and conducted the study. The following licenses/restrictions apply: the author can analyze the raw data and prepare any report/article/presentation. Requests to access these datasets should be directed to PARPA, parpa@parpa.pl.

## Ethics Statement

The studies involving human participants were reviewed and approved by The Maria Grzegorzewska University Ethics Committee. Written informed consent for participation was not required for this study in accordance with the national legislation and the institutional requirements.

## Author Contributions

MR designed the study, analysed statistically the data, and prepared the first draft of the manuscript in full.

## Conflict of Interest

The author declares that the research was conducted in the absence of any commercial or financial relationships that could be construed as a potential conflict of interest.

## Publisher’s Note

All claims expressed in this article are solely those of the authors and do not necessarily represent those of their affiliated organizations, or those of the publisher, the editors and the reviewers. Any product that may be evaluated in this article, or claim that may be made by its manufacturer, is not guaranteed or endorsed by the publisher.
