# Supplementary material for: Differences and Similarities in Motives to Decrease Drinking, and to Drink in General Between Former and Current Heavy Drinkers—Implications for Changing Own Drinking Behaviour
Source: Front Psychol. 2022 Jan 12;12:734350. doi: 10.3389/fpsyg.2021.734350 (PMC8789672; doi:10.3389/fpsyg.2021.734350)
Supplement: Supplementary file 1 [file Table_1.DOCX]

Supplementary Material

Supplementary Table 1. Drinking motives models’ comparison

| model | Chi^2^ | CFI | TLI | Standardised MRM | RMSEA |
| --- | --- | --- | --- | --- | --- |
| 4 factor – original model | Chi^2^(47)= 152, p<.001 | .952 | .943 | .050 | .068 |
| 3 factor (11-item) model | Chi^2^(40)=122, p<.001 | .96 | .945 | .048 | .065 |
| 3 factor (9-item) model | Chi^2^(24)=71.98, p<.001 | .971 | .957 | .041 | .064 |

Supplementary Table 2. Convergent and discriminant validity, correlational matrix with the square root of the AVE on the diagonal

|  | **CR** | **AVE** | **MSV** | **MaxR(H)** | **SocEnh** | **Coping** | **Conformity** |
| --- | --- | --- | --- | --- | --- | --- | --- |
| **SocEnh** | 0,763 | 0,519 | 0,407 | 0,777 | **0,720** |  |  |
| **Coping** | 0,801 | 0,574 | 0,407 | 0,804 | 0,638 | **0,758** |  |
| **Conformity** | 0,834 | 0,627 | 0,253 | 0,843 | 0,401 | 0,503 | **0,792** |

Supplementary Table 3. CFA for motives to decrease drinking

| Because… | Factorial loadings | | |
| --- | --- | --- | --- |
|  | 1 | 2 | 3 |
| I lost control over my drinking | .924 |  |  |
| People close to me expected me do limit my drinking (family, friends, partner) | .892 |  |  |
| When I was under the influence of alcohol, I did something I regrated or was ashamed of | .770 |  |  |
| It interfered with my work / school / other duties | .650 |  |  |
| I outgrew it |  | .882 |  |
| I moved out / finished studies |  | .861 |  |
| In order to get rid of physical negative effects of drinking |  |  | .905 |
| In order to get rid of psychological negative effects of drinking |  |  | .622 |
| *Cronbach’s alpha* | *.662* | *.518* | *.712* |

*Annotation: PCA was applied with Oblimin rotation due to intercorrelation of factors*

Supplementary Table 4. CFA for RALD

| Because… | Factor loadings | | |
| --- | --- | --- | --- |
|  | 1 | 2 | 3 |
| it’s not healthy to drink too much | .885 |  |  |
| it could interfere with my carrying out my responsibilities (like school work, my job). | .865 |  |  |
| I feel physically ill after drinking | .841 |  |  |
| the people I hang around with are against drinking | .830 |  |  |
| I’m scared I’ll get into trouble | .692 |  |  |
| I sometimes become rude or obnoxious when I drink |  | .932 |  |
| it’s against my religion to drink |  | .841 |  |
| it’s expensive |  | .490 |  |
| I’m afraid I might become an alcoholic if I drink too much |  |  | .939 |
| I worry that I might not be able to control myself. |  |  | .444 |
